# Low-Cost Alternative External Rotation Shoulder Brace and Review of Treatment in Acute Shoulder Dislocations

**DOI:** 10.5811/westjem.2014.12.23068

**Published:** 2015-01-06

**Authors:** Kyle Lacy, Chris Cooke, Pat Cooke, Justin Schupbach, Rahul Vaidya

**Affiliations:** * Detroit Medical Center, Department of Orthopaedic Surgery, Detroit, Michigan; † Wayne State University, Detroit Medical Center, Detroit, Michigan

## Abstract

Traumatic dislocations of the shoulder commonly present to emergency departments (EDs). Immediate closed reduction of both anterior and posterior glenohumeral dislocations is recommended and is frequently performed in the ED. Recurrence of dislocation is common, as anteroinferior labral tears (Bankart lesions) are present in many anterior shoulder dislocations.^14^,^15^,^18^,^23^ Immobilization of the shoulder following closed reduction is therefore recommended; previous studies support the use of immobilization with the shoulder in a position of external rotation, for both anterior and posterior shoulder dislocations.^7^–^11^,^19^ In this study, we present a technique for assembling a low-cost external rotation shoulder brace using materials found in most hospitals: cotton roll, stockinette, and shoulder immobilizers. This brace is particularly suited for the uninsured patient, who lacks the financial resources to pay for a pre-fabricated brace out of pocket. We also performed a cost analysis for our low-cost external rotation shoulder brace, and a cost comparison with pre-fabricated brand name braces. At our institution, the total materials cost for our brace was $19.15. The cost of a pre-fabricated shoulder brace at our institution is $150 with markup, which is reimbursed on average at $50.40 according to our hospital billing data. The low-cost external rotation shoulder brace is therefore a more affordable option for the uninsured patient presenting with acute shoulder dislocation.

## INTRODUCTION

The acute traumatic shoulder dislocation is a frequent reason for presentation to emergency departments (EDs). Anterior dislocations compose up to 96% of all shoulder dislocations, and often result from excessive external rotation with the shoulder in a position of abduction and external rotation.^13^,^25^ Posterior dislocations are less frequent, and may result from an excessive traumatic posterior force with the shoulder in internal rotation, flexion, and adduction.^19^ Injury mechanisms for posterior shoulder dislocation include motor vehicle collision, fall, seizure, electrocution, and sports-related injury.^1^,^19^ Immediate closed reduction of all shoulder dislocations is recommended, and is often performed in the ED. Anteroinferior labral tears (Bankart lesions) are present in many anterior shoulder dislocations, and contribute to instability and recurrent dislocation.^14^,^15^,^18^,^23^ The presence of a large Hill-Sachs defect or reverse Hill-Sachs defect (>1.5cm^3^) also correlates with recurrent dislocation.^19^ Age, sex, and athletic activity also contribute to recurrence, with higher rates of recurrent dislocation and need for surgical stabilization seen in younger patients, athletes, and male patient groups.^3^,^4^,^14^,^15^,^18^,^22^,^23^ In acute traumatic shoulder dislocation, instability is seen in 19–67%, recurrence of dislocation in 15–57%, and immediate immobilization is therefore recommended.^4^,^12^,^15^,^18^

Over time, recurrent shoulder dislocations lead to higher rates of arthropathy.^3^,^5^ Physical therapy after a period of immobilization is recommended, though motion restriction bands designed to avoid stretching the anteroinferior capsule have not been shown to reduce recurrence.^11^,^22^

Posterior dislocations are immobilized in external rotation or a “gunslinger” position of neutral rotation, abduction, and slight flexion.^19^ The position of immobilization for anterior shoulder dislocations is somewhat controversial. External rotation tightens the anterior capsule and subscapularis tendon, which pull the medially displaced labroligamentous complex from the glenoid neck back up onto the rim; cadaveric studies have verified this coaptation effect as well as increased glenohumeral contact force with external rotation.^7^,^16^ Magnetic resonance imaging studies of patients with anterior dislocations have confirmed this coaptation effect on the torn anteroinferior labrum, as well as a reduction in anterior capsule volume with external rotation.^8^,^12^,^20^,^21^,^23^ Itoi et al., in a randomized controlled trial of 40 patients (average age of 37 years) with anterior shoulder dislocation, showed a recurrence rate of 30% with conventional internal rotation immobilization, and zero dislocations with external rotation immobilization.^9^ A second study of 198 patients randomized for three weeks of immobilization in either internal or external rotation, showed a recurrent dislocation relative risk reduction of 38.2% in favor of external rotation.^10^ In a study of 33 patients with acute primary anterior dislocation comparing external and internal rotation immobilization, Taskoparan, et. al., found lower rates of recurrent dislocation with external rotation, which were significant in the 21–30 year age group.^24^ However, larger randomized controlled trials, as well as meta-analyses comparing external and internal rotation immobilization for acute traumatic shoulder dislocation, have not shown a statistically significant difference in regards to recurrence of dislocation.^2^,^15^,^17^,^26^,^27^ Although controversial, an external rotation shoulder brace can be used for both anterior and posterior shoulder dislocations.

At our institution, many of our patients lack medical insurance, or the financial resources necessary to pay for a prefabricated external rotation shoulder brace. Expensive shoulder braces are therefore not an option for many of our patients. This impetus has led us to develop a low-cost alternative shoulder immobilizer brace, using materials found in most hospitals. The following is a technique guide for assembling a low-cost alternative external rotation shoulder brace.

## TECHNIQUE

**Figure 1:**
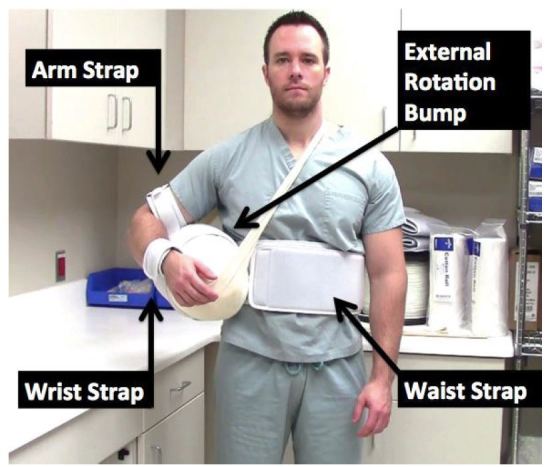
The assembled low-cost external rotation shoulder brace. The four components consist of the waist strap, the arm strap, the wrist strap, and the external rotation bump.

**Figure 2:**
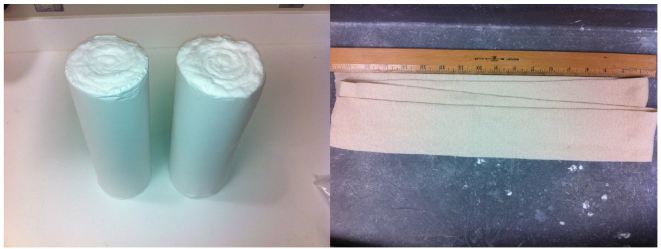
Two 14-inch cotton rolls and 4 feet of 4 inch stockinette are prepared.

**Figure 3:**
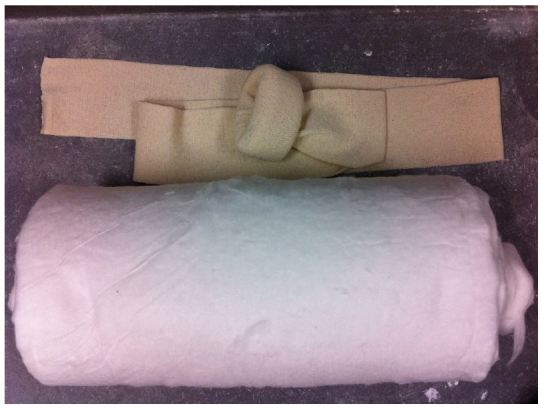
The stockinette is cuffed, to allow for insertion of the cotton roll.

**Figure 4:**
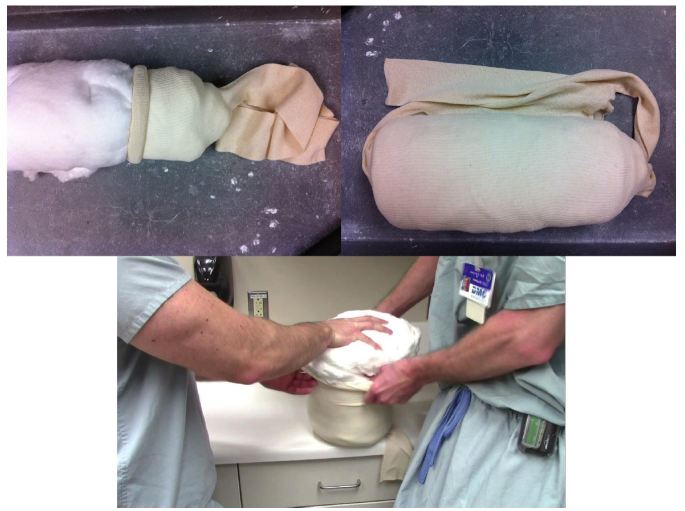
The cotton roll is inserted into the stockinette.

**Figure 5:**
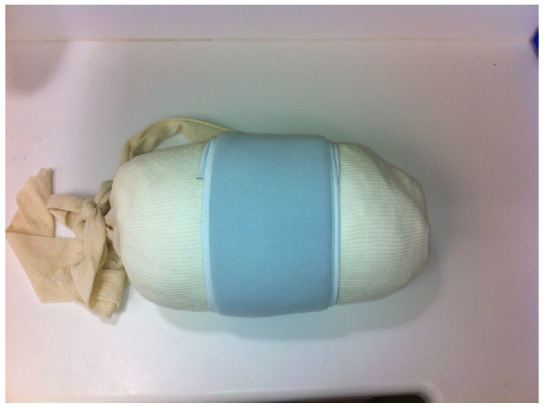
A waist strap is wrapped around the bump and fastened, completing the external rotation bump.

The low-cost external rotation shoulder brace consists of four components: the waist strap, the arm strap, the wrist strap, and the external rotation bump (Figure 1, Video). Materials for the external rotation shoulder brace include: two 14” practical cotton rolls, four feet of 4” stockinette roll (Figure 2), two standard shoulder immobilizer sets. To make the external rotation bump, the two 14” cotton rolls are rolled together into one thicker roll. The 4” stockinette is then rolled in on itself in a cuff-like fashion, leaving roughly two feet free of the cuff (Figure 3). An end of the cotton roll is then stuffed into the cuffed opening of the stockinette, for a snug fit. The stockinette cuff is then rolled over the cotton roll, engulfing it entirely (Figure 4). A waist strap from the first shoulder immobilizer set is then cut to size, wrapped around the cotton-stuffed stockinette, and fastened. This serves as the external rotation bump (Figure 5). The cotton within the bump can be molded with compression over the posterior aspect, so as to create the desired degree of external rotation.

**Figure 6:**
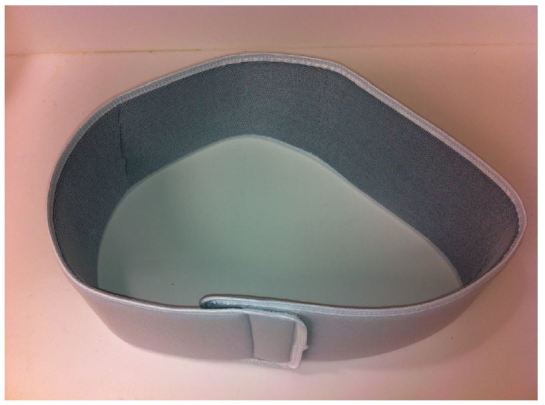
The waist strap.

**Figure 7:**
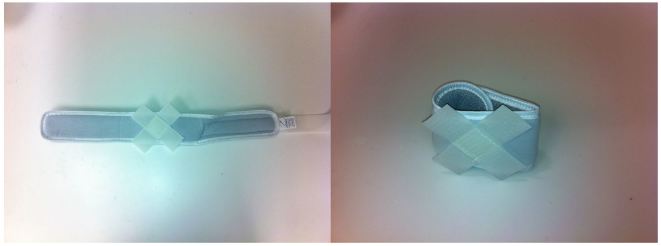
The arm strap and the slightly smaller wrist strap.

**Figure 8:**
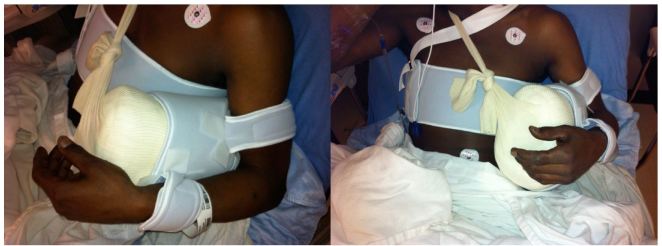
The ideal shoulder immobilization position of 10 degrees of external rotation and 20 degrees of abduction.

The waist strap from the shoulder immobilizer (Figure 6) is then fitted around the patient’s waist and fastened. The arm strap is placed around the patient’s arm, while the slightly shorter wrist strap (Figure 7) is placed around the patient’s wrist; both are fastened. The fasteners of these straps then adhere to the foam exterior of the external rotation bump, thus supporting the arm. The free stockinette ends of the external rotation bump are then tied around the patient’s neck. The external rotation bump can be further molded, to an ideal shoulder position of 10 degrees of external rotation, and 20 degrees of abduction (Figure 8).

We obtained itemized materials cost information for the low-cost brace from our hospital’s operative room billing data. Additionally, the cost of a pre-fabricated external rotation shoulder brace at our institution was also obtained; this included our institutional mark-up as well as the average payer reimbursement for the pre-fabricated brace. These numbers represent the price our institution pays to the supplier, and therefore accounts for cost discounts attributed to economies of scale, as these materials are purchased in bulk. We obtained the prices for eight different brand-name prefabricated external rotation shoulder braces through a simple search on Amazon.com using the criteria “external rotation shoulder brace.”

## RESULTS

Material costs for the supplies needed to construct an external rotation shoulder immobilizer are listed in Table 1. The total material cost for our external rotation shoulder brace = 2 standard shoulder immobilizers ($10.58) + stockinette 4” × 4’ ($2.21) + 2 practical cotton rolls ($6.36) = $19.15. Our hospital is contracted with DJO Global (Vista, CA) for the Donjoy® Ultrasling™ shoulder braces; price for the braces with mark-up included at our institution was $150 per brace; Medicare reimbursement for each brace was quoted at $50.40, as only roughly one-third of the total cost is reimbursed to the hospital. The listed prices on Amazon.com of eight different brand-name prefabricated external rotation shoulder braces are listed in Table 2. Throughout this search, we did not find a prefabricated external rotation shoulder brace whose listed price was lower than the total materials cost of our brace. In comparison, the average Internet-listed price for a prefabricated shoulder brace was 4.6 times that of the total materials cost of our brace.

## DISCUSSION

The low-cost alternative external rotation shoulder brace is useful for the acute immobilization of the reduced anterior or posterior shoulder dislocation. This low-cost brace can be easily assembled in the ED using materials commonly found in most hospitals. An advantage to this brace is that assembly only takes a few minutes, and can be performed by on-call residents or attending physicians at any hour of the day. The brace therefore does not require the assistance of an orthotics vendor or technician for fitting and sizing, which is also an advantage. At our institution, the orthopaedic surgery resident on-call fits the brace, and therefore this creates no additional cost. In the event that an uninsured patient cannot afford to pay for a shoulder brace out of pocket, our hospital then absorbs the cost. Therefore, the brace offers the potential for cost savings to both the patients and the hospital.

Anterior dislocations are commonly associated with anteroinferior labral tears (Bankart lesions), which often displace medially onto the glenoid neck. The resultant loss of the bumper effect created by the anteroinferior labrum leads to recurrent instability (subluxation or dislocation) of the humeral head off the anterior glenoid. In both cadaveric and human studies, a position of shoulder external rotation has been shown to have a “coaptation effect” on the anteroinferior labral tear. Tension of the subscapularis and anterior capsule in external rotation reduces the capsular volume, and mobilizes the displaced anteroinferior labral tear off the medial glenoid neck, thus reducing it back up onto the anterior glenoid rim.^7^,^8^,^12^,^16^,^20^,^21^,^23^ Randomized clinical outcomes studies have shown reduced rates of recurrent shoulder dislocation after immobilization in external rotation.^9^,^10^,^24^ However, larger randomized controlled trials and meta-analyses comparing positions of internal and external rotation for shoulder immobilization following reduction after shoulder dislocation have shown no difference in rates of dislocation recurrence.^2^,^15^,^17^,^26^,^27^ Although a controversial topic, immobilization of the shoulder in a position of external rotation is a safe and effective treatment option for both anterior and posterior shoulder dislocations.

## LIMITATIONS

We did not compare the clinical efficacy of our brace with that of the brand-name prefabricated external rotation shoulder braces. Rates of recurrence of shoulder dislocation with the use of this low-cost brace were not assessed, nor was the comparative durability of our brace assessed. Additionally, we did not assess ease of use and patient satisfaction with our brace. The listed materials cost of our low-cost external rotation shoulder brace is only directly applicable to our institution (Detroit Receiving Hospital); the materials cost may differ among hospitals due to differences in patient volume and economies of scale. In spite of these limitations, for uninsured patients at our institution who cannot afford to pay for a brace out-of-pocket, this low-cost alternative brace is often the preferred option.

## CONCLUSION

For the self-pay patient without adequate funds to pay for a shoulder brace out of pocket, our low-cost external rotation brace is a useful alternative. The external rotation brace can be fitted to create the desired degree of abduction and external rotation. The brace can be easily assembled using materials found in most hospitals, and can assist in the immobilization of patients with acute anterior and posterior shoulder dislocations at a fraction of the cost.

## Figures and Tables

**Video:** Low-cost alternative external rotation shoulder brace assembly technique guide.

**Table 1:** Itemized materials cost for the low-cost external rotation shoulder brace.

Item	Cost
2 Shoulder immobilizers	$10.58
Stockinette 4″x4′	$2.21
2 Cotton rolls	$6.36
Total shoulder brace materials cost	$19.15

**Table 2:** Itemized materials cost for the low-cost external rotation shoulder brace.

Item	Cost	Source
Donjoy® Ultrasling™ DRH Cost	$50.40	Detroit Receiving or Billing
Donjoy® Ultrasling™ with Markup	$150.00	
Breg® Neutral Wedge Shoulder Brace	$136.99	Amazon.com
Donjoy® Ultrasling™ III ER 30in	$89.00	
Donjoy® Ultrasling™ II	$75.00	
Donjoy® Ultrasling™ III X-Large	$114.99	
Corflex® Shoulder Abduction Pillow Sling	$69.99	
Corflex® ER Shoulder Abduction Pillow with Sling	$79.99	
Maxar® AS-300™ Super Arm Sling	$104.75	
AlphaBrace® Shoulder Immobilizer and Sling	$35.65	
Average shoulder brace price	$88.30	
